# Reconstruction of Toll-like receptor 9-mediated responses in HEK-Blue hTLR9 cells by transfection of human macrophage scavenger receptor 1 gene

**DOI:** 10.1038/s41598-017-13890-3

**Published:** 2017-10-20

**Authors:** Shozo Ohtsuki, Yuki Takahashi, Takao Inoue, Yoshinobu Takakura, Makiya Nishikawa

**Affiliations:** 1 Department of Biopharmaceutics and Drug Metabolism, Graduate School of Pharmaceutical Sciences, Kyoto University, Sakyo-ku, Kyoto, 606-8501 Japan; 2 Division of Molecular Target and Gene Therapy Products, National Institute of Health Sciences, Setagaya-ku, Tokyo, 158-8501 Japan; 3 Laboratory of Biopharmaceutics, Faculty of Pharmaceutical Sciences, Tokyo University of Science, Noda, Chiba, 278-8510 Japan

## Abstract

We used human Toll-like receptor 9 (hTLR9)-expressing HEK-Blue hTLR9 cells, which release secreted embryonic alkaline phosphatase (SEAP) upon response to CpG DNA, to evaluate the immunological properties of nucleic acid drug candidates. Our preliminary studies showed that phosphodiester CpG DNA hardly induced any SEAP secretion in HEK-Blue hTLR9 cells. In the current study, therefore, we developed HEK-Blue hTLR9 cells transduced with human macrophage scavenger receptor-1 (hMSR1), a cell-surface DNA receptor, and determined whether HEK-Blue hTLR9/hMSR1 cells respond to phosphorothioate (PS) CpG DNA and phosphodiester (PO) CpG DNA. We selected PS CpG2006, a single-stranded PO CpG DNA (ssCpG), and a tetrapod-like structured DNA (tetrapodna) containing ssCpG (tetraCpG) as model TLR9 ligands. Alexa Fluor 488-labeled ligands were used for flow cytometry. Unlike the mock-transfected HEK-Blue hTLR9 cells, the HEK-Blue hTLR9/hMSR1 cells efficiently took up all three CpG DNAs. SEAP release was almost proportional to the uptake. Treatment of HEK-Blue hTLR9/hMSR1 cells with an anti-hMSR1 antibody significantly reduced the uptake of ssCpG and tetraCpG. Collectively, reconstruction of TLR9-mediated responses to CpG DNA in HEK-Blue hTLR9 cells can be used to evaluate the toxicity of nucleic acid drug candidates with diverse physicochemical properties.

## Introduction

Various classes of nucleic acid drugs have been marketed or are being developed. Attention must be paid to toxicity issues during the development of nucleic acid drugs^1^. Nucleic acid drug candidates have several toxicity issues, including off-target effects, immune stimulation, hematoxicity, hepatotoxicity, and nephrotoxicity. Immune stimulation occurs when toll-like receptors (TLRs) recognize DNA or RNA. Several reports have suggested that certain small interfering RNA molecules cause immune stimulation via TLRs^2–8^. Therefore, it is clearly important to evaluate unintentional TLR-mediated immune stimulation in the development of nucleic acid drug candidates.

TLR9 is the only TLR that recognizes DNA. Its ligand is a DNA molecule containing an unmethylated cytosine–phosphate–guanine (CpG) motif, *i.e*., CpG DNA. Because bacterial and viral DNAs contain many CpG motifs, TLR9-mediated responses form part of the self-defense system against the invasion of such pathogens^9–11^. TLR9 is expressed in the endosomes of various mammalian cells, including plasmacytoid dendritic cells and B-cells^12^. After cellular uptake and sorting into endosomes, CpG DNA binds to TLR9 and induces the release of proinflammatory cytokines, which then activate innate immunity^13,14^. It is expected that such responses can be exploited to treat cancer, infection, and allergic diseases^15,16^. We have previously reported that nano-construction of CpG DNA is a unique and promising method of increasing the immunostimulatory activity of CpG DNA^17^. Studies using a series of polypod-like structured DNAs demonstrated that the cellular uptake of CpG DNA was significantly increased by its incorporation into nanostructured DNAs^18–20^. However, the mechanisms involving the structure-dependent uptake of DNA require further elucidation.

The endosomal localization of TLR9 indicates that membrane protein(s) other than TLR9 are responsible for the cellular uptake of DNA. So far, several DNA receptors have been reported. These include macrophage scavenger receptor-1 (MSR1, SR-A, and CD204), α_M_β_2_ (MAC-1), advanced glycosylation end product-specific receptor (AGER), membrane-associated nucleic acid-binding protein (MNAB), mannose receptor-1 (MRC1), and lymphocyte antigen 75 (DEC-205)^21–26^. However, in most cases their contribution to DNA uptake has been examined using phosphorothioate (PS) DNA, which non-specifically binds to cell membranes more strongly than natural phosphodiester (PO) DNA. The results obtained with PS DNA cannot be used to estimate the contribution of DNA receptors in the cellular responses to nucleic acid drug candidates. Msr1, which mediates the endocytosis of negatively charged molecules such as acetylated low-density lipoprotein (LDL) and oxidized LDL, is reportedly involved in the uptake of PO DNA by macrophages^21^. Therefore, Msr1-mediated DNA uptake could be involved in TLR-mediated immune stimulation by nucleic acid drug candidates.

HEK-Blue TLR cells are commercially available and can be used for the analysis of the immunological properties of various TLR ligands^27^. HEK-Blue hTLR9 cells respond to PS CpG DNA and release secreted embryonic alkaline phosphatase (SEAP)^28^. However, our preliminary studies have shown that PO CpG DNA induces very little SEAP release from HEK-Blue hTLR9 cells. Because HEK293 cells, which constitute the parent cell line of HEK-Blue TLR9 cells, are non-immune cells, they do not express any DNA receptors on their cell membranes. Therefore, we hypothesized that low cellular uptake of CpG DNA by HEK-Blue hTLR9 cells might explain the weak or absent response to PO CpG DNA. Therefore, in the present study, we sought to establish cell lines that respond to both PS and PO CpG DNAs. Such cells would be useful for the screening of nucleic acid drug candidates with diverse physicochemical properties. To this end, we transduced HEK-Blue hTLR9 cells with human *MSR1* to obtain HEK-Blue hTLR9/hMSR1 cells in the hope that the transfection of the *MSR1* gene to HEK-Blue hTLR9 cells would increase the uptake of PO DNA. We first evaluated the effect of transfection of the *MSR1* gene on the cellular uptake of DNA. We then determined whether HEK-Blue hTLR9/hMSR1 cells respond to both PS and PO CpG DNAs. We selected phosphorothioate CpG2006 (PS CpG2006), a single-stranded PO CpG DNA (ssCpG), and a tetrapod-like structured DNA containing the ssCpG (tetraCpG) as model TLR9 ligands. HEK-Blue hTLR9 cells and HEK-Blue hTLR7 cells were also used for the analysis of cellular responses to CpG DNA.

## Results

### Establishment of HEK-Blue hTLR9/hMSR-1 Cells

**Figure 1:**
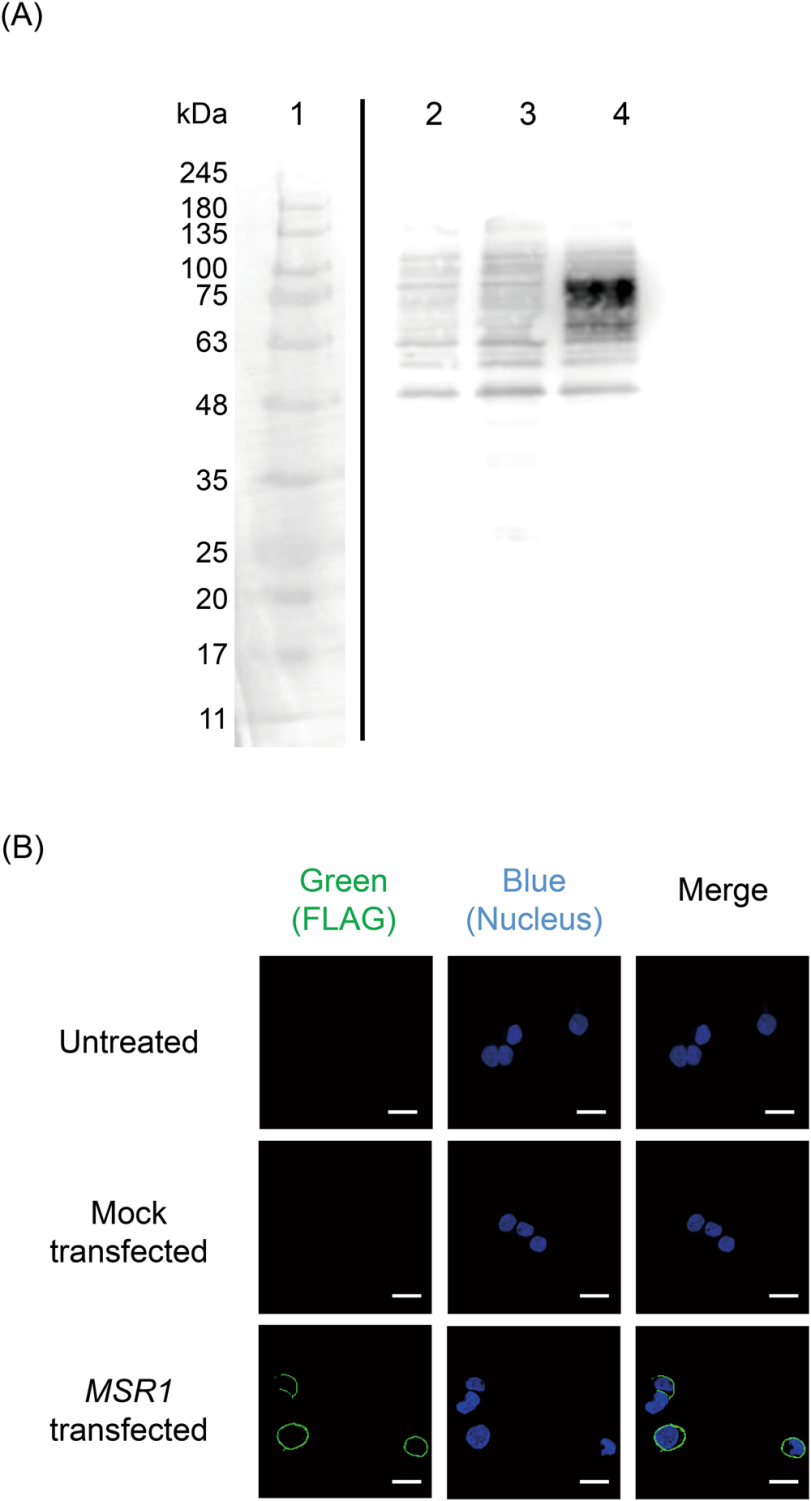
Confirmation of hMSR1 expression in HEK-Blue hTLR9 cells. (**A**) hMSR-1 protein was detected by western blotting using anti-hMSR1 antibody. Bright field (lane 1) and chemiluminescence (lanes 2–4) images were shown. Lane 1, protein size marker; lane 2, untreated HEK-Blue hTLR9 cells; lane 3, mock-transfected HEK-Blue hTLR9 cells; lane 4, HEK-Blue hTLR9/hMSR1 cells. The full-size and low-contrast images of the gel are shown in Supplementary Fig. S1. (**B**) Confocal microscopy images of untreated, mock-transfected, or *MSR1*-transfected HEK-Blue hTLR9 cells immunostained with anti-FLAG antibody and Alexa Fluor-488 conjugated anti-mouse IgG1 antibody. Scale bar, 20 μm.

Figure 1A shows the results of western blotting analysis of the cell lysates using anti-hMSR1 antibody. The lysate of HEK-Blue hTLR9/hMSR1 cells revealed a band of approximately 75 kDa, which corresponded to the FLAG-tagged hMSR1. The band was not detected in the lysates of the untreated or mock-transfected HEK-Blue hTLR9 cells, indicating that FLAG-tagged hMSR1 was expressed in the HEK-Blue hTLR9/hMSR1 cells. We examined the localization of hMSR1 in the HEK-Blue hTLR9 cells using confocal microscopy. Figure 1B presents confocal microscopy images of untreated, mock-transfected, and *MSR1*-transfected HEK-Blue hTLR9 cells. The Alexa Fluor 488-labeled anti-FLAG antibody was bound to the cell surface of the HEK-Blue hTLR9/hMSR1 cells. However, there was little fluorescence in the untreated or mock-transfected cells. These results indicate that hMSR1 localized at the cell surface of the HEK-Blue hTLR9/hMSR1 cells.

### Evaluation of the functions of HEK-Blue hTLR9/hMSR-1 cells

**Figure 2:**
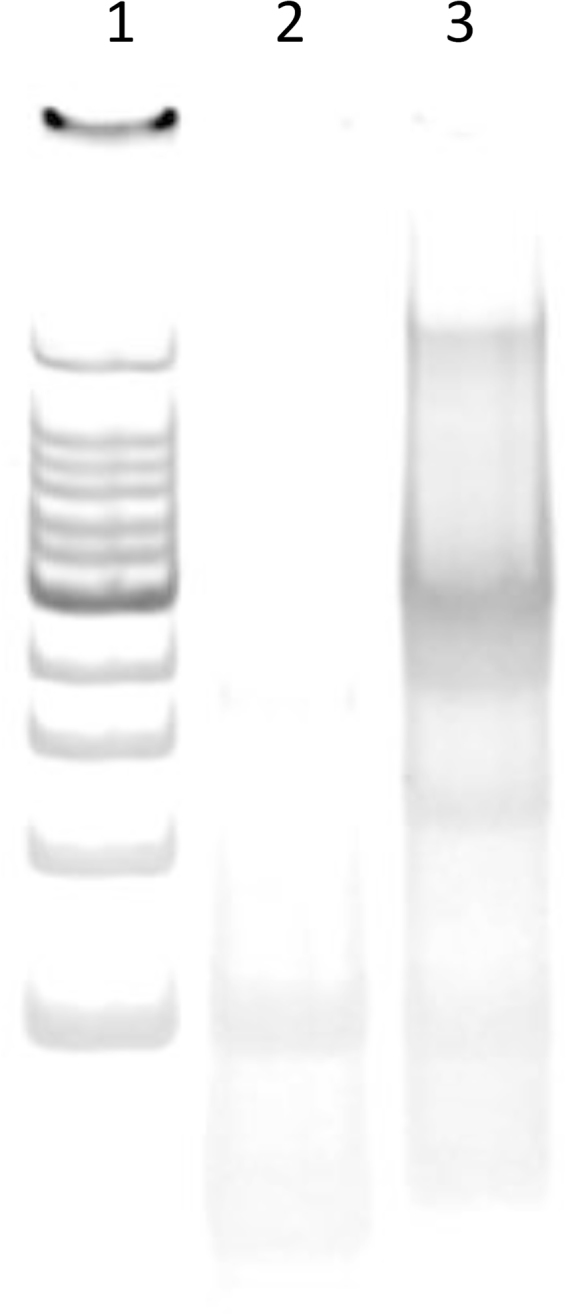
Electrophoretic analysis of ssCpG and tetraCpG. Aliquots of ssCpG and tetraCpG were run on 6% PAGE at room temperature. Lane 1, 100 bp ladder; lane 2, ssCpG; lane 3, tetraCpG. The full-size and low-contrast image of the gel is shown as Supplementary Fig. S2.

Figure 2 shows the results of the PAGE analysis of ssCpG and tetraCpG prepared at a DNA concentration of 100 μM. ssCpG and tetraCpG are represented by single PAGE bands, indicating that tetraCpG had been prepared with high efficiency.

**Figure 3:**
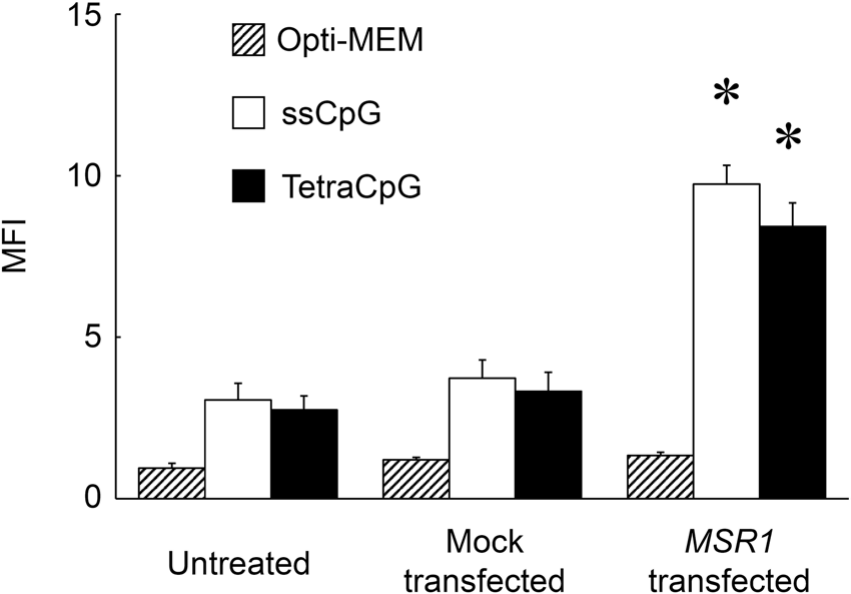
Uptake of ssCpG and tetraCpG in untreated, mock-transfected, or *MSR1-*transfected HEK-Blue hTLR9 cells. Each Alexa Fluor 488-labeled DNA sample was added to cells at a concentration of 2 μg/mL. The results are expressed as means + SEM of three independent experiments. *P < 0.05 compared with the mock-transfected group.

Figure 3 shows the MFI of the HEK-Blue hTLR9 cells, the mock-transfected HEK-Blue hTLR9 cells, and the HEK-Blue hTLR9/hMSR1 cells after the addition of Alexa Fluor 488-labeled DNA samples. The MFI values of the HEK-Blue hTLR9/hMSR1 cells were significantly higher than those of the mock-transfected HEK-Blue hTLR9 cells after addition of Alexa Fluor 488-ssCpG or tetraCpG. There was no significant difference in the MFI value of the HEK-Blue hTLR9/hMSR1 cells between Alexa Fluor 488-ssCpG and tetraCpG. In contrast, the MFI values of the cells after addition of Alexa Fluor 488-PS CpG2006 were not significantly different among the cells, irrespective of hMSR1 expression (data not shown).

**Figure 4:**
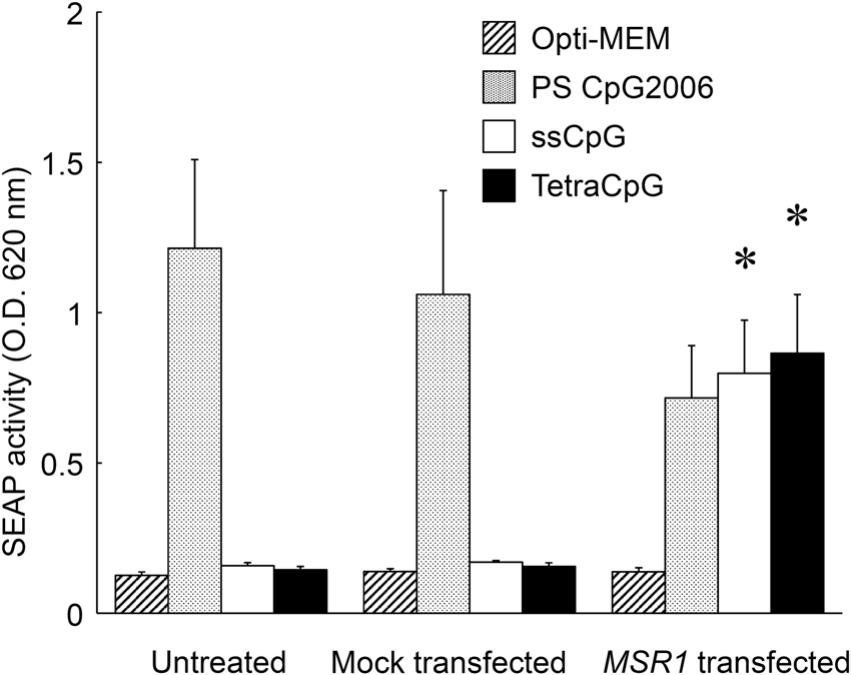
Secreted embryonic alkaline phosphatase (SEAP) release from untreated, mock-transfected, or *MSR1-*transfected HEK-Blue hTLR9 cells using HEK-Blue detection solution. Each DNA sample was added to the cells at a final concentration of 50 μg/mL, and the OD of the sample was measured at 620 nm. All oligodeoxynucleotides (ODNs) have a phosphodiester backbone except for CpG2006. The results are expressed as means + SEM of three independent experiments. *P < 0.05 compared with the mock-transfected group.

Figure 4 shows SEAP activity after the addition of PS CpG2006, ssCpG, and tetraCpG. The HEK-Blue hTLR9 cells or mock-transfected HEK-Blue hTLR9 cells released SEAP upon the addition of PS CpG2006, but did not release SEAP after the addition of ssCpG or tetraCpG. However, PS CpG2006, ssCpG, and tetraCpG all induced SEAP release from the HEK-Blue hTLR9/hMSR1 cells. There was no significant difference in SEAP activity after the addition of PS CpG2006 among the three types of cells. The SEAP activities of the HEK-Blue hTLR9/hMSR1 cells were comparable with each other after the addition of ssCpG and tetraCpG.

**Figure 5:**
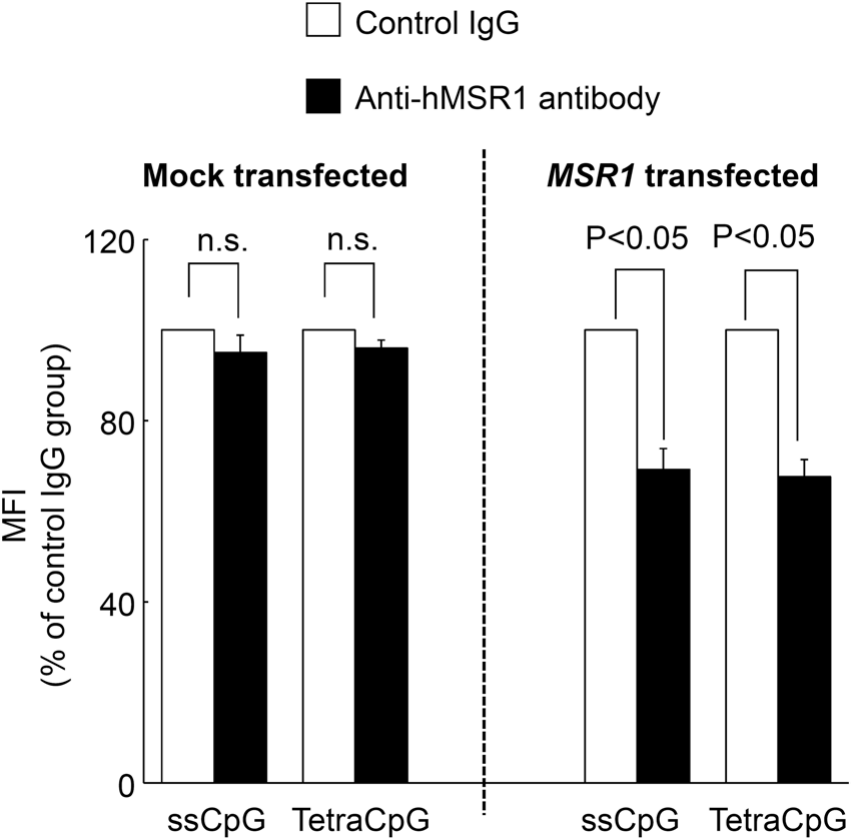
Cellular uptake of ssCpG and tetraCpG in mock-transfected or *MSR1-*transfected HEK-Blue hTLR9 cells in the presence of mouse IgG1 isotype control or hMSR1 antibody. Each Alexa Fluor 488-labeled DNA sample was added to cells at a final concentration of 2 μg/mL. The results are expressed as means + SEM of four independent experiments.

The uptake of Alexa Fluor 488-ssCpG and tetraCpG was examined in the presence of anti-hMSR1 antibody to confirm the involvement of hMSR1 in the uptake of DNA by HEK-Blue hTLR9/hMSR1 cells. Figure 5 shows the MFI of mock-transfected HEK-Blue hTLR9 cells and HEK-Blue hTLR9/hMSR1 cells after the addition of Alexa Fluor 488-labeled DNA samples in the presence of hMSR1 antibody or murine IgG1 isotype control antibody. Anti-hMSR1 antibody significantly reduced the uptake of Alexa Fluor 488-ssCpG and tetraCpG in the HEK-Blue hTLR9/hMSR1 cells.

### Experiments using HEK-Blue hTLR7 cells

**Figure 6:**
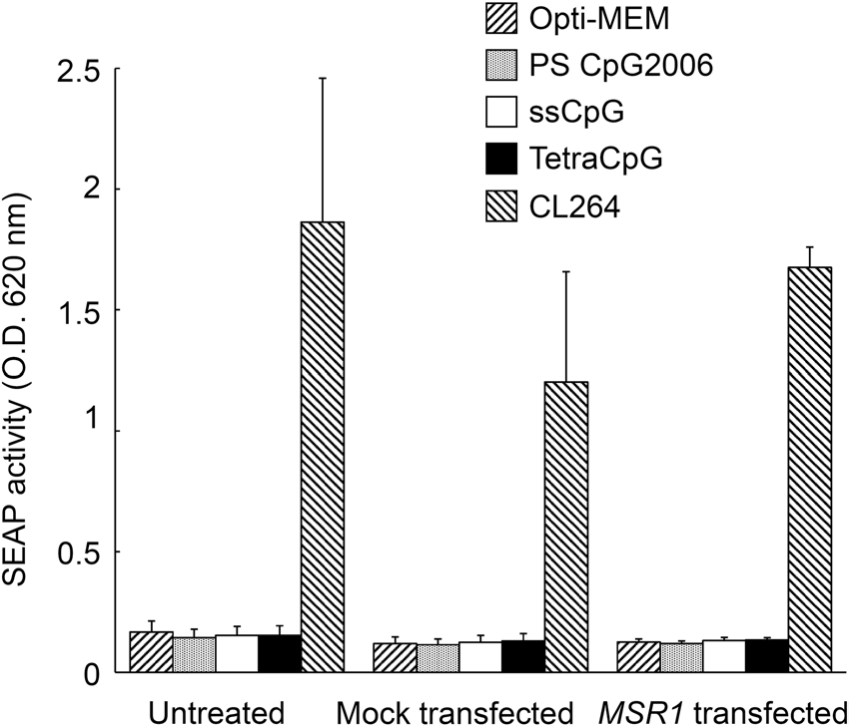
Secreted embryonic alkaline phosphatase (SEAP) release from untreated, mock-transfected, or *MSR1-*transfected HEK-Blue hTLR7 cells using a HEK-Blue detection solution. Each DNA sample was added to the cells at a final concentration of 50 μg/mL. The sample optical density (OD) was measured at 620 nm. The results are expressed as means + SEM of three independent experiments. CL264; a TLR7 ligand.

To exclude the possibility that the expression of hMSR1 results in SEAP release without recognition of CpG DNA by TLR9, HEK-Blue hTLR7 cells were used instead of HEK-Blue hTLR9. The expression of hMSR1 in HEK-Blue hTLR7/hMSR1 cells was confirmed by western blotting (data not shown). Figure 6 shows SEAP activity after the addition of PS CpG2006, ssCpG, and tetraCpG to HEK-Blue hTLR7 cells. CL264, a TLR7 ligand, induced significant SEAP release. In contrast, PS CpG2006, ssCpG, and tetraCpG scarcely induced SEAP release from the HEK-Blue hTLR7 cells, irrespective of the expression of hMSR1. These results indicate that SEAP is released from HEK-Blue hTLR9/hMSR1 cells through the recognition of CpG DNA by TLR9.

## Discussion

In the present study, we demonstrated that HEK-Blue hTLR9 cells efficiently responded to PS CpG DNA, which has high binding affinity for cell membranes, whereas they hardly responded to natural, PO CpG DNA, in spite of the fact that the cells expressed human TLR9. We also found that the low cellular uptake of PO CpG DNA by HEK-Blue hTLR9 cells explains their limited response to PO CpG DNA, and that transformation of the cells with a plasmid expressing hMSR1, a DNA receptor, restored the response of the cells to PO CpG DNA. Therefore, HEK-Blue hTLR9/hMSR1 cells can be used as a sensitive screening system for compounds that activate TLR9, although it could be difficult using HEK-Blue hTLR9/hMSR1 cells to discuss the physiological processes of the interaction of CpG DNA with TLR9-expressing cells or the mechanistic details of the cellular uptake of DNA.

The results of the present study suggest that hMSR1 or other DNA receptors are not expressed in HEK-Blue hTLR9 or HEK293 cells. HEK293 cells, the parental cell line of HEK-Blue hTLR9 cells, have been widely used for the transfection of genes because they facilitate easy transformation. Therefore, HEK-Blue hTLR9 cells are also suitable for transfection studies. Both HEK293 cells and HEK-Blue hTLR9 cells hardly take up PO DNA, so they can be used to explore the receptors responsible for DNA binding, especially PO DNA binding.

MSR1 is a membrane protein that is located on the cell surface of macrophages and dendritic cells^29,30^. We found that the hMSR1 expressed in HEK-Blue hTLR9/hMSR1 cells also localized at the cell membrane (Fig. 1B). The *MSR1* cDNA used in the present study contained hMSR1 signal-anchor sequences, so it is reasonable to assume that hMSR1 is appropriately sorted to the correct destination (the cell membrane). It has been reported that ligation to MSR1 induces clathrin-mediated endocytosis, and that the ligands are then sorted to endosomes^31^. The mechanistic details of the uptake of DNA by HEK-Blue hTLR9/hMSR1 cells were not investigated in this study, but the efficient response to PO CpG DNA strongly suggests that the cells take up DNA in a similar manner to that adopted by other types of cells that express MSR1, such as dendritic cells.

Several reports suggest that hMSR1 is involved in the cellular uptake of PS CpG DNA^32^. However, the present study demonstrated that hMSR1 expression had no significant effect on the cellular uptake of Alexa Fluor 488-PS CpG DNA (data not shown) or on SEAP release (Fig. 4). PS CpG DNA binds strongly to cell surfaces^33^, and would mask any hMSR1-mediated cellular uptake of PS CpG DNA, even if it occurred.

Although most TLR9 is found in on the endosomes, TLR9 is also detected on the surface of cells in some cell types^34,35^. Some reports discussed that the cell surface TLR9 promoted the cellular uptake of CpG DNA as well as CpG DNA-coupled siRNA^36–38^. In these studies, PS CpG DNA and the antisense strand of siRNA were conjugated and, therefore, a strong binding of PS CpG DNA to the cell surface could lead to efficient uptake of the conjugate. Zhang *et al*. demonstrated that the cell surface TLR9 did not participate in the uptake of CpG DNA^37^. Therefore, the cell surface TLR9 on HEK-Blue hTLR9/hMSR1 cells, even if it exists, would not be critical for the immune responses to PO CpG DNA.

Our previous studies demonstrated that RAW264.7, DC2.4, and bone marrow-derived dendritic cells (BMDCs) took up nanostructured DNAs more efficiently than single-stranded or double-stranded DNAs^17–20,39^. In the present study, we showed that hMSR1 can recognize nanostructured DNAs, such as tetrapodna. However, no significant differences were observed in the uptake by HEK-Blue hTLR9/hMSR1 cells between Alexa Fluor 488-ssCpG and Alexa Fluor 488-tetraCpG. These differences suggest that cell surface receptors other than MSR1 or auxiliary molecules are involved in the efficient cellular uptake of DNAs with complicated structures.

Taken together, the results of the present study demonstrate that the reconstruction of toll-like receptor 9-mediated responses to CpG DNA in HEK-Blue hTLR9 cells is useful for evaluating and predicting the TLR9-dependent toxicity of nucleic acid drug candidates, irrespective of their physicochemical properties. Our results also suggest that the combination of HEK-Blue hTLR9 cells and natural PO CpG DNA can be used to screen DNA receptors or DNA-binding proteins on the cell surface. Additional studies on other cell surface DNA receptors will improve our understanding of the mechanisms underlying the interactions between DNA and cells at the molecular level.

## Methods

### Chemicals

Dulbecco’s modified Eagle’s medium (DMEM) was obtained from Nissui Pharmaceutical, Co., Ltd. (Tokyo, Japan). Sodium chloride, sodium hydrogen phosphate, sodium bicarbonate, potassium chloride, glucose, sodium dodecyl sulfate (SDS), methanol, and WIDE-VIEW Prestained Protein Size Marker III were purchased from Wako Pure Chemicals Industries, Ltd. (Osaka, Japan). Tris was obtained from Nacalai Tesque (Kyoto, Japan). Blasticidin, zeocin, normocin, CL264, and HEK-Blue detection reagents were purchased from InvivoGen (San Diego, CA, USA). Opti-modified Eagle’s medium (Opti-MEM) and fetal bovine serum (FBS) were obtained from Thermo Fisher Scientific Inc. (Waltham, MA, USA). A 100-base pair (bp) DNA ladder was purchased from Takara Bio (Otsu, Japan). All other chemicals were of the highest grade available and were used without further purification.

### Cell Culture

HEK-Blue hTLR7 and HEK-Blue hTLR9 cells were obtained from InvivoGen. The cells were cultured in DMEM supplemented with 10% heat-inactivated FBS, 0.2% sodium bicarbonate, 100 IU/mL penicillin, 100 μg/mL streptomycin, 2 mM l-glutamine, 30 μg/mL blasticidin, 100 μg/mL zeocin, and 100 μg/mL normocin at 37 °C in humidified air containing 5% CO_2_ as per the manufacturer’s instructions.

### Plasmid DNA

Plasmid pcDNA3.1 was purchased from Thermo Fisher Scientific Inc. A plasmid vector encoding human macrophage scavenger receptor-1 (human MSR1, hSR-A, hCD204) was constructed by insertion of the FLAG-tagged *MSR1* fragment amplified by polymerase chain reaction (PCR) from a cDNA clone of human MSR1 (GE Healthcare UK Ltd., Buckinghamshire, England) into the multi-cloning site of pcDNA3.1.

### Transfection of hMSR1-expressing Plasmid DNA in HEK-Blue Cells

HEK-Blue hTLR7 and HEK-Blue hTLR9 cells were cultured in 75-cm^2^ tissue culture flasks, and were transfected with a pcDNA3.1 vector encoding *MSR1* or an empty pcDNA3.1 vector using Lipofectamine 2000 (Thermo Fisher Scientific Inc.) according to the manufacturer’s instructions. After 20 h of incubation, the cells were used as HEK-Blue hTLR7/hMSR1 and HEK-Blue hTLR9/hMSR1 cells. The cells transfected with empty pcDNA3.1 vector were used as mock controls.

### Western Blotting of hMSR1 in HEK-Blue hTLR Cells

The cells were lysed in a lysis buffer (PicaGene Dual Sea Pansy Luminescence Kit, Toyo Ink, Tokyo, Japan), and the cell lysates were reduced by the addition of dithiothreitol to 100 mM. A fraction of the cell lysate (7 μg protein) was subjected to 10% SDS-polyacrylamide gel electrophoresis (PAGE) and transferred to a polyvinylidene fluoride transfer membrane (Immobilon-P; Merck Millipore Ltd, Darmstadt, Germany). The membrane was then blocked in Blocking One (Nacalai Tesque, Kyoto, Japan). The membrane was incubated with anti-hMSR1 antibody (R&D Systems, Minneapolis, MN, USA) for 1 h at 20–22 °C. The membrane was then incubated with horseradish peroxidase (HRP)-conjugated rabbit anti-mouse IgG antibody (Thermo Fisher Scientific Inc.) for 1 h at room temperature. Protein bands were detected by chemiluminescence using an Immobilon Western chemiluminescent HRP substrate (Merck Millipore, Billerica, MA, USA).

### Confocal Microscopic Detection of hMSR1 in HEK-Blue Cells Transduced with hMSR1

Untreated, mock-transfected, or *MSR1*-transfected HEK-Blue hTLR9 cells were seeded on a chamber slide at a density of 3 × 10^4^ cells/well and then cultured for 24 h. The cells were washed twice with phosphate-buffered saline (PBS), fixed with 4% paraformaldehyde for 20 min, and washed again twice with PBS. The cells were then blocked with 20% FBS in PBS for 1 h. The cells were incubated with anti-FLAG M2 antibody (Sigma-Aldrich, St. Louis, MO, USA) and 10% FBS in PBS for 1 h at room temperature, and then washed once. The cells were incubated with Alexa Fluor 488-labeled anti-mouse IgG antibody (Abcam Plc, Cambridge, UK) for 1 h at room temperature, and washed once. The cells were incubated with 600 nM 4′,6-diamidino-2-phenylindole (DAPI; Life Technologies) for 5 min at room temperature and washed once. The chamber was then removed and the slide was observed using a confocal microscope (A1R MP, Nikon Instech Co., Ltd., Tokyo, Japan) as previously reported^39^.

### Oligodeoxynucleotides

All oligodeoxynucleotides (ODNs) used were purchased from Integrated DNA Technologies, Inc. (Coralville, IA, USA). The sequences of the ODNs used are presented in Table 1. Phosphodiester ODN-1, which contained a potent human CpG motif (GTCGTT), was used as single-stranded CpG DNA (ssCpG). ssCpG and three other phosphodiester ODNs were dissolved in an annealing buffer (TE buffer, 10 mM Tris-HCl, pH 8, 1 mM ethylenediaminetetraacetic acid, and 150 mM sodium chloride) and mixed in sterile water to produce a final concentration of 100 μM for each ODN. The mixtures were then incubated at 95 °C for 5 min and slowly cooled to 4 °C using a thermal cycler to obtain tetrapodna containing ssCpG (tetraCpG). Phosphorothioate CpG2006 (PS CpG2006), a single-stranded B-type CpG DNA, was used as a positive control to induce SEAP release from the HEK-Blue hTLR9 cells. For cellular uptake experiments, ODN-1 labeled with Alexa Fluor 488 at the 5′ end was purchased from Japan BioService Co., Ltd. (Saitama, Japan). Each sample was analyzed at room temperature by 6% PAGE. The DNA bands were visualized using SYBR Gold (Molecular Probes, Eugene OR, USA).

**Table 1:** The sequences of the oligodeoxynucleotides (ODNs) for the DNA nanostructures.

Name	Sequences (5′ → 3′)	Length (mer)
ODN-1	TCGTCGTTTTGTCGTTTTGTCGTTTACATTCCTAAGTCTGAAACATTACAGCTTGCTACACGAGAAGAGCCGCCATAGTA	80
ODN-1′	TACTATGGCGGCTCTTCTCGTGTAGCAAGCTGTAATGTTTCAGACTTAGGAATGT	55
tetrapodna-2	TTACTATGGCGGCTCTTCTCGTGTAGCATAGTGTCGTTTTATCACCAGGCAGTTG	55
tetrapodna-3	TCAACTGCCTGGTGATAAAACGACACTACGTGGGAATCTTGACAGGTCATCAGCC	55
tetrapodna-4	TGGCTGATGACCTGTCAAGATTCCCACGAGCTGTAATGTTTCAGACTTAGGAATG	55
PS CpG2006	T*C*G*T*C*G*T*T*T*T*G*T*C*G*T*T*T*T*G*T*C*G*T*T	24

All ODNs have a phosphodiester backbone. The asterisks (*) indicate the positions of phosphorothioate (PS) modifications. The CpG motif (GTCGTT) is underlined.

### Uptake of DNA in HEK-Blue Cells

Untreated, mock-transfected, or *MSR1*-transfected HEK-Blue hTLR9 cells were seeded onto 48-well plates at a density of 1 × 10^5^ cells/well. Alexa Fluor 488-ssCpG or Alexa Fluor 488-tetraCpG diluted with 0.1 mL of Opti-MEM was then added to the cells. After 2 h incubation at 37 °C, the cells were washed three times with 400 μL of PBS and harvested. The fluorescence intensity of the cells was then determined by flow cytometry (Gallios Flow Cytometer; Beckman Coulter, Inc., CA, USA) using Kaluza software (version 1.0; Beckman Coulter), and the mean fluorescence intensity (MFI) was calculated. Similar experiments were carried out on the untreated, mock-transfected, and HEK-Blue hTLR7 cells.

### SEAP release from HEK-Blue Cells

The untreated, mock-transfected, or *MSR1*-transfected HEK-Blue hTLR9 cells were seeded onto 96-well plates at a density of 5 × 10^4^ cells/well. PS CpG2006, ssCpG, or tetraCpG in HEK-Blue detection solution was added to the cells to produce a final concentration of 50 μg/mL. After 20 h incubation at 37 °C, the optical density (OD) of the samples was measured at a wavelength of 620 nm using a microplate reader. Similar experiments were carried out on untreated, mock-transfected, and HEK-Blue hTLR7 cells.

### Inhibition of DNA Uptake in HEK-Blue Cells by Anti-hMSR1 Antibody

The untreated, mock-transfected, or *MSR1*-transfected HEK-Blue hTLR9 cells were seeded onto 48-well plates at a density of 1 × 10^5^ cells/well. The cells were pretreated with anti-hMSR1 antibody or IgG1 isotype control (R&D Systems, Minneapolis, MN, USA) at a concentration of 2 μg/mL for 1 h. After washing, the cells were treated with 2 μg/mL Alexa Fluor 488-ssCpG or Alexa Fluor 488-tetraCpG diluted with 0.1 mL of Opti-MEM together with anti-hMSR1 antibody or IgG1 isotype control to produce a final concentration of 2 μg/mL. After 2 h incubation at 37 °C, the cells were washed three times with 400 μL of PBS and harvested. The fluorescence intensity of the cells was determined by flow cytometry using Kaluza software, and the MFIs were calculated.

### Statistical Analysis

Differences were evaluated statistically by one-way analysis of variance (ANOVA), followed by the Tukey–Kramer test for multiple comparisons and the Student’s t-test for two groups. *P* < 0.05 values were considered statistically significant.

## Electronic supplementary material

Supplementary Information
