# Supplementary material for: Reconstruction of Toll-like receptor 9-mediated responses in HEK-Blue hTLR9 cells by transfection of human macrophage scavenger receptor 1 gene
Source: Sci Rep. 2017 Oct 20;7:13661. doi: 10.1038/s41598-017-13890-3 (PMC5651873; doi:10.1038/s41598-017-13890-3)
Supplement: Supplementary file 1 — Supplementary Information [file 41598_2017_13890_MOESM1_ESM.pdf]

Reconstruction of Toll-like receptor 9-mediated responses in HEK-Blue hTLR9 cells by transfection of human macrophage scavenger receptor 1 gene

Shozo Ohtsuki[1], Yuki Takahashi[1], Takao Inoue[2], Yoshinobu Takakura[1] and Makiya Nishikawa[1,3,*]

[1] Department of Biopharmaceutics and Drug Metabolism, Graduate School of Pharmaceutical Sciences, Kyoto University, Sakyo-ku, Kyoto 606-8501, Japan
[2] Division of Molecular Target and Gene Therapy Products, National Institute of Health Sciences, Setagaya-ku, Tokyo 158-8501, Japan
[3] Laboratory of Biopharmaceutics, Faculty of Pharmaceutical Sciences, Tokyo University of Science, Noda, Chiba 278-8510, Japan

* To whom correspondence should be addressed. Tel: +81-4-7121-4450; Fax: +81-4-7121-4450; Email: makiya@rs.tus.ac.jp

Supplementary Information

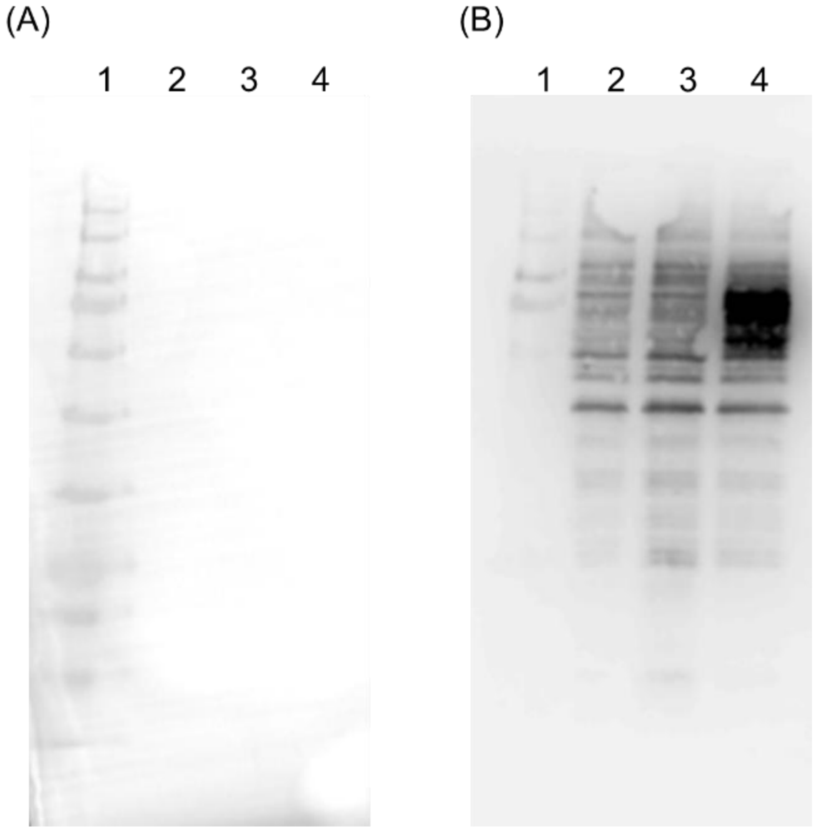

Supplementary Figure S1. Full-size and low-contrast images of the gel. Bright field (A) and chemiluminescence (B) images were shown. Lane 1, protein size marker; lane 2, untreated HEK-Blue hTLR9 cells; lane 3, mock-transfected HEK-Blue hTLR9 cells; lane 4, HEK-Blue hTLR9/hMSR1 cells.

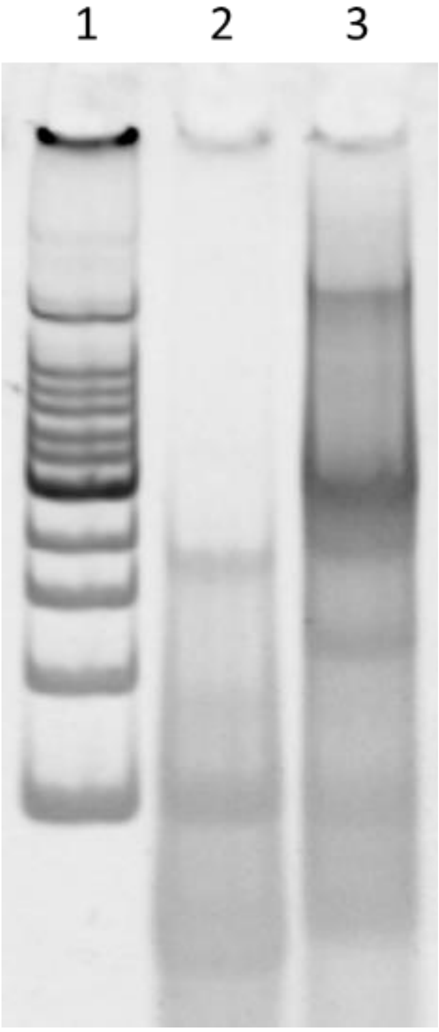

Supplementary Figure S2. Full-size and low-contrast image of the gel. Lane 1, 100 bp ladder; lane 2, ssCpG; lane 3, tetraCpG.
